# P-1520. Addition of Relebactam Increases Susceptibility to Imipenem Among Imipenem-Intermediate *Pseudomonas aeruginosa*: Asia/Pacific SMART 2018-2022

**DOI:** 10.1093/ofid/ofae631.1689

**Published:** 2025-01-29

**Authors:** Mark G Wise, C Andrew DeRyke, Wei-Ting Chen, Fakhar Siddiqui, Katherine Young, Mary Motyl, Daniel F Sahm

**Affiliations:** IHMA, Schaumburg, Illinois; IHMA, Schaumburg, Illinois; MSD Taiwan, Taipei, Taipei, Taiwan; Merck & Co., Inc., Rahway, New Jersey; Merck, Rahway, New Jersey; Merck, Rahway, New Jersey; IHMA, Schaumburg, Illinois

## Abstract

**Background:**

Imipenem/relebactam (IMR) combines imipenem with the β-lactamase inhibitor relebactam, an inhibitor of class A and C β-lactamases. We assessed the activity of IMR and comparators against *Pseudomonas aeruginosa* collected in 9 countries in the Asia/Pacific region as part of the global SMART surveillance program, with a focus on the increased antimicrobial activity brought by the addition of relebactam to imipenem for those organisms interpreted as “intermediate” by 2024 CLSI criteria (MIC = 4 µg/mL) to imipenem alone.

**Figure:**
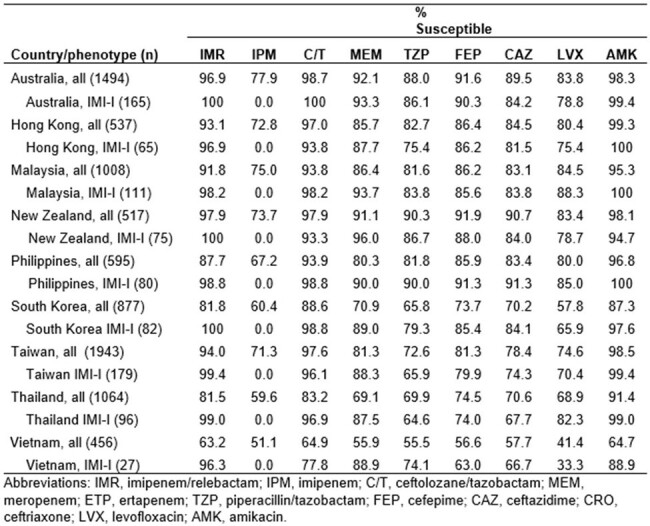

**Methods:**

From 2018-2022, 50 clinical laboratories in nine countries in Asia/Pacific (Australia, Hong Kong, Malaysia, New Zealand, Philippines, South Korea, Taiwan, Thailand, and Vietnam) each collected up to 250 consecutive, aerobic or facultative, Gram-negative pathogens per year from patients with bloodstream, intraabdominal, lower respiratory tract, and urinary tract infections. MICs were determined using CLSI broth microdilution and interpreted with 2024 CLSI breakpoints. Most isolates that were imipenem or ceftolozane/tazobactam-nonsusceptible (NS) were screened for β-lactamases.

**Results:**

IMR demonstrated excellent antimicrobial activity against isolates from most countries, inhibiting >80% of the isolates from each country except Vietnam (63.2% susceptible; Table). Ceftolozane/tazobactam and amikacin showed similar levels of activity. Limiting the analysis to isolates that tested “intermediate” to imipenem (MIC = 4 µg/mL) revealed that the addition of relebactam rendered >96% of the organisms from each country susceptible, ranging from 100% (Australia, New Zealand and South Korea) to 96.3% (Vietnam). Most imipenem-I *P. aeruginosa* examined molecularly (727/740; 98.2%) did not carry an acquired β-lactamase. As relebactam itself does not possess antibacterial activity, this suggests that the increased antimicrobial activity attributable to its addition is likely a result of its inhibitory activity on the intrinsic AmpC (*bla*_PDC_) in this species.

**Conclusion:**

IMR showed potent activity against clinical *P. aeruginosa* collected in Asia/Pacific. The addition of relebactam to imipenem was responsible for the inhibition of >96% of isolates that were “intermediate” to imipenem alone.

**Disclosures:**

**Daniel F. Sahm, PhD**, Pfizer, Inc.: Advisor/Consultant

